# Elevated pretreatment platelet-to-lymphocyte ratio is associated with poor survival in stage IV non-small cell lung cancer with malignant pleural effusion

**DOI:** 10.1038/s41598-019-41289-9

**Published:** 2019-03-18

**Authors:** Jeong Uk Lim, Chang Dong Yeo, Hye Seon Kang, Chan Kwon Park, Ju Sang Kim, Jin Woo Kim, Seung Joon Kim, Sang Haak Lee

**Affiliations:** 1 Division of Pulmonary, Critical Care and Sleep Medicine, Department of Internal Medicine, College of Medicine, The Catholic University of Korea, Seoul, Republic of Korea; 2 Cancer Research Institute, College of Medicine, The Catholic University of Korea, Seoul, Republic of Korea

## Abstract

A higher platelet-to-lymphocyte ratio (PLR) has a clinical correlation with shorter survival in non-small cell lung cancer (NSCLC). The present study evaluated the association between the PLR and survival in patients with advanced NSCLC with malignant pleural effusion (MPE). Between January 2012 and July 2016, 237 patients with stage IV NSCLC were selected for evaluation. Receiver operating characteristic analysis was used to determine a cutoff for the PLR. Clinicopathological characteristics were compared between the high and low PLR groups, and the role of PLR as a predictive/prognostic maker was investigated. Among the 237 patients, 122 were assigned to the low PLR group and the other 115 to the high PLR group. According to multivariate analysis, male sex, not receiving active anticancer treatment, low hemoglobin level, low albumin level, high C-reactive protein level, and high PLR were identified as significant risk factors for shorter overall survival (OS) (*p* = 0.010, <0.001, 0.011, 0.004, 0.003, and <0.001, respectively). In the subgroup multivariate analysis of driver mutation-negative NSCLC, high Eastern Cooperative Oncology Group score, not receiving active anticancer treatment, low hemoglobin level, high C-reactive protein level, and high PLR were identified as significant risk factors for shorter OS (*p* = 0.047, <0.001, = 0.036, = 0.003, and <0.001, respectively). A high pretreatment PLR is independently associated with poor survival in stage IV NSCLC with MPE and in a subgroup of epidermal growth factor receptor and anaplastic lymphoma kinase wild-type NSCLC.

## Introduction

Lung cancer is one of the leading causes of cancer death worldwide^1^. Non-small cell lung cancer (NSCLC) comprises 85% of all lung cancers^2^. Despite recent advances in treatment modalities, such as targeted therapies, the 5-year survival rate of NSCLC is only 16.6%^3^.

Inflammation plays a key role in cancer progression^4^. Several studies have shown that elevated systemic inflammation contributes to angiogenesis, invasion, and metastasis of cancer cells^5,6^. Furthermore, an association between inflammation and poor prognosis has been confirmed in various cancers^7–9^. The clinical significance and usefulness of inflammatory markers that can predict prognosis during cancer treatment are increasing^10^.

In lung cancer, inflammatory markers such as C-reactive protein (CRP), neutrophil-to-lymphocyte ratio, Glasgow prognostic score, and platelet-to-lymphocyte ratio (PLR) have been evaluated^11–13^. Among them, PLR, which is defined as the absolute platelet count divided by the absolute lymphocyte count, is a widely studied inflammatory marker^10,14^. Its prognostic value has been evaluated in various types of cancer, including colorectal, breast, ovarian, and pancreatic^15–17^. In NSCLC, an elevated PLR is associated with poor overall survival (OS) and progression-free survival (PFS)^3,14^. An elevated PLR was also associated with poor prognosis in surgically treated NSCLC patients^13^. Pretreatment PLR was identified as an independent risk factor for brain metastasis of lung adenocarcinoma^10^. Although the predictive and prognostic values of PLR have been evaluated in both advanced lung cancer and lung cancer requiring surgery, PLR has not been assessed specifically in stage IV NSCLC patients with cytologically proven malignant pleural effusion (MPE). MPE is common complication which can occur during the course of treatment in advanced NSCLC^18,19^. Moreover, NSCLC patients with MPE show poor prognosis with a median survival time of less than 12 months^20^, and complain more respiratory symptoms which lead to decreased quality of life^21^. While they show distinct and more unfavorable clinical manifestations, evaluation of prognostic values of PLR in this subgroup of advanced NSCLC is meaningful.

Furthermore, the prognostic and predictive values of PLR in patients with epidermal growth factor receptor (EGFR) and anaplastic lymphoma kinase (ALK) wild-type NSCLC specifically have not been investigated. The evaluation of PLR in a driver mutation-negative subgroup can be clinically important after excluding patients with EGFR and ALK mutations, who are likely to have improved survival^22^. Patients with EGFR mutations are eligible for targeted therapy and thus have a longer median survival compared with those without EGFR mutations^22^. For patients with an ALK gene rearrangement, the ALK tyrosine kinase inhibitor crizotinib is an available treatment modality^23^. In addition, crizotinib therapy improved the survival of patients with ALK-positive NSCLC compared with those not treated with crizotinib^23^.

In this study, we evaluated the predictive and prognostic values of PLR in stage IV NSCLC patients with MPE and characterized the clinical significance of PLR in a wild-type EGFR and ALK NSCLC subgroup.

## Methods

### Patient selection

Among a cohort of patients with NSCLC, a total of 237 patients with stage IV NSCLC with MPE were consecutively selected. They were diagnosed with NSCLC between January 2012 and July 2016 and were enrolled from six university hospitals: Yeouido St. Mary’s Hospital, Seoul St. Mary’s Hospital, Bucheon St. Mary’s Hospital, Incheon St. Mary’s Hospital, St. Paul’s Hospital, and Uijeongbu St. Mary’s Hospital^24^. Inclusion criteria for enrollment were patients with 1) MPE diagnosed either by pleural biopsy that proved malignant or cytologically and 2) all clinical data available from electronic medical record. Exclusion criteria were patients 1) with SCLC, 2) with significant infection, 3) who were on antibiotic treatment at the time of enrollment, and 4) with a concurrent hematologic disease^24^.

### Clinical and laboratory data

For all enrolled patients, data including sex, age, histology, tumor stage by tumor–node–metastasis stage (AJCC 2009), Eastern Cooperative Oncology Group performance status (ECOG PS), smoking status, and baseline blood chemistry values were collected for all enrolled patients. The PLR was calculated based on the initial pretreatment complete blood count.

### EGFR and ALK testing

Excluding other uncommon EGFR profiles, EGFR mutations were defined as an exon 21 point mutation or an exon 19 deletion. EGFR genotyping was performed by peptide nucleic acid (PNA)-mediated PCR clamping methods using the PNAClamp^TM^ EGFR Mutation Detection Kit (PANAGENE, Inc., Daejeon, Korea)^24,25^.

Specimens for FISH acquired from the study centers were prepared using a molecular analysis platform and analyses were performed at Yeouido St. Mary’s Hospital Central Molecular Laboratory^24,26^. Utilizing a break-apart probe specific to the ALK locus, the Vysis LSI ALKDual Color Break Apart Probe (Abbott Molecular, Abbott Park, IL, USA), FISH was carried out on formalin-fixed paraffin-embedded (FFPE) tumor specimens. Positive ALK rearrangement was defined as an isolated red signal or a split signal; those with >15% of counted nuclei within tumor cells exhibiting an isolated red signal or split signal were considered as positive cases. A specimen-specific assessment approach was used to minimize technical bias. A total of 100 tumor cells were scored in surgical resection specimens. An ALK FISH split signal rate of <15% was interpreted as negative^26^.

### Chemotherapy and adverse reactions

Systemic conventional chemotherapies given to the patients comprised pemetrexed, docetaxel, gemcitabine, or paclitaxel combined with cisplatin/carboplatin regimens^24^. Targeted therapies were comprised of erlotinib, gefitinib, and afatinib for positive EGFR mutations and crizotinib for positive ALK translocations. As a first line regimen, the standard recommendation in the study centers was platinum-based doublet chemotherapy regimen, however in some special cases, monotherapy regimen was also considered for treatment.

During the treatment course, all study patients were checked for treatment-related adverse reactions either at the outpatient clinic or during admission. All significant adverse reactions were available from electronic hospital records. Treatment was either delayed or changed to another regimen, when patients experienced grade III or IV adverse reactions.

### OS and PFS

Response Evaluation Criteria in Solid Tumors version 1.1 was used to evaluate responses to cancer treatment^27^. For response evaluation, patients underwent a computed tomography scan after every two cycles of treatment. Treating physicians and independent radiologists evaluated the responses. OS was defined as the time from the date of lung cancer diagnosis to death. PFS was defined as the time duration between lung cancer diagnosis and disease progression after first-line treatment. Patients were considered censored if they died or lost contact during the follow-up period^24^.

### Defining cutoff values

The optimal cutoff value for PLR was determined by receiver operating characteristic (ROC) analysis. Survival outcomes were dichotomized by survival (alive or dead). All survival statuses were assessed in October 2017.

For other continuous values entered in the univariate analyses of clinical outcomes, their cutoff points were also calculated from ROC curves. Normal range values were used if the p-value from the ROC analysis was not significant.

### Statistical analysis

All statistical analyses were done using the Statistical Package for Social Sciences software version 18.0 (SPSS Inc., Chicago, IL, USA). Data of continuous variables are shown as means or medians with ranges. The Chi-squared test was performed to compare categorical parameters. Continuous variables were analyzed using two-sided t-tests or the Mann–Whitney U test depending on the distribution status.

Univariate analysis using the Cox regression model was performed to determine the variables significantly associated with OS and PFS. The median OS and PFS are presented as 95% confidence intervals. Survival curves were constructed using Kaplan–Meier analysis. A log-rank test was performed to determine significant differences in the survival outcomes between groups. Statistically significant variables were entered into multivariate analysis using the Cox proportional hazards regression model. A *P* value less than 0.05 was considered statistically significant in all analyses.

### Ethics statement

The present study was approved by the ethics committee of the hospitals: Seoul St. Mary’s Hospital, Incheon St. Mary’s Hospital, Yeouido St. Mary’s Hospital, Bucheon St. Mary’s Hospital, St. Paul’s Hospital, and Uijeongbu St. Mary’s Hospital (XC17REDI0069U). The need for informed consent was waived by the institutional review boards.

## Results

### Patient characteristics

The baseline characteristics of the 237 patients with NSCLC are summarized in Table 1. Their median age was 69 (range, 32–92) years, and 132 (55.7%) patients were males. The majority of patients were diagnosed with adenocarcinoma (86.9%) and/or had an ECOG PS of 0/1 (84.4%). A total of 222 patients underwent active anticancer treatment, including 152 (64.1%) who underwent first-line conventional systemic chemotherapy and 70 (29.5%) who underwent targeted therapy; only 15 (6.3%) patients did not undergo active anticancer treatment and received supportive care. Among 152 patients who underwent first-line conventional systemic chemotherapy, 145 patients received plantinum-based doublet chemotherapy regimen, while only 7 patients underwent monotherapy. EGFR mutations were found in 87 (36.7%) patients. The median OS was 15.4 (range, 11.9–18.9) months. The median PFS after first-line treatment was 6.9 (range, 5.7–8.1) months.

**Table 1:** Baseline clinical characteristics of patients.

	Overall Patients (*N*, %)	Low PLR (N, %)	High PLR (*N*, %)	p-value*
Number of patients	237	122	115	
Median age, range	69 (32–92)	67 (38–92)	70 (32–90)	
Median OS (months), 95% CI	15.4 (11.9–18.9)	28.3 (19.7–36.9)	10.7 (7.0–14.4)	<0.001
Median PFS (months), 95% CI	6.9 (5.7–8.1)	8.1 (7.9–9.0)	5.6 (4.5–6.8)	0.092
Sex				0.440
Male	132 (55.7)	65 (53.3)	67 (58.3)	
Female	105 (44.3)	57 (46.7)	48 (41.7)	
ECOG				0.708
0 and 1	200 (84.4)	104 (85.2)	96 (83.5)	
≥2	37 (15.6)	18 (14.8)	19 (16.5)	
Histologic features				0.127
Adenocarcinoma	206 (86.9)	110 (90.2)	96 (83.5)	
Squamous	31 (13.1)	12 (9.8)	19 (16.5)	
Smoking				0.992
Never smoker	131 (55.3)	68 (55.7)	63 (55.3)	
Ever smoker	105 (44.3)	54 (44.2)	51 (44.7)	
T-factor				0.551
T1/T2/T3/T4	11 (4.6)/30 (12.7)/31 (13.1)/98 (41.4)	7 (8.0)/17 (19.5)/13 (14.9)/50 (57.5)	4 (4.8)/13 (15.7)/18 (21.7)/48 (57.8)	
N-factor				0.644
N0/N1/N2/N3	13 (5.5)/12 (5.1)/45 (19.0)/99 (41.8)	8 (9.5)/5 (6.0)/20 (23.8)/51 (60.7)	5 (5.9)/7 (8.2)/25 (29.4)/48 (56.5)	
M-factor				0.446
M1a/M1b	110 (46.4)/97 (40.9)	58 (55.8)/46 (44.2)	52 (50.5)/51 (49.5)	
Treatment modality (1^st^ line)				0.926
Conventional chemotherapy	152 (64.1)	79 (64.8)	73 (63.5)	
Platinum-based doublet	145 (95.4)	76 (96.2)	69 (94.5)	
Targeted therapy	70 (29.5)	36 (29.5)	34 (29.6)	
Supportive care	15 (6.3)	7 (5.7)	8 (7.0)	
Pleurodesis	52 (21.9)	31 (27.9)	21 (19.8)	0.161
EGFR mutation	87 (36.7)	45 (39.8)	42 (40.8)	0.886
ALK mutation	12 (5.1)	7 (6.9)	5 (5.2)	0.626
WBC count (x10^9^/L)	8966.7 ± 4121.0	8807.9 ± 2899.6	9135.2 ± 5116.4	0.542
Hemoglobin (g/dL)	13.2 ± 1.7	13.7 ± 1.6	12.7 ± 1.6	<0.001
Platelet (per/uL)	298,440 ± 78,947	269,040 ± 64,321	329,630 ± 81,273	<0.001
Platelet-to-lymphocyte ratio	207.4 ± 131.0	135.0 ± 28.8	284.3 ± 151.8	<0.001
CRP (mg/L)	18.3 ± 34.2	14.4 ± 31.1	22.5 ± 36.9	0.076
CEA (ng/mL)	142.8 ± 561.5	114.2 ± 615.0	511.2 ± 62.0	0.581
LDH	504.3 ± 250.4	496.4 ± 211.4	512.6 ± 286.6	0.624
Protein	6.6 ± 0.7	6.7 ± 0.7	6.6 ± 0.7	0.704
Albumin	3.7 ± 0.5	3.7 ± 0.5	3.6 ± 0.5	0.005

ALK: anaplastic lymphoma kinase; BMI: body mass index; CEA: carcinoembryonic antigen; CRP: c-reactive protein; CI: confidence interval; ECOG: Eastern Cooperative Oncology Group; EGFR: epidermal growth factor receptor; LDH: lactate dehydrogenase; OS: overall survival; PFS: progression free survival; WBC: white blood cell.

*p-value between high PLR group and low PLR group.

**Figure 1:**
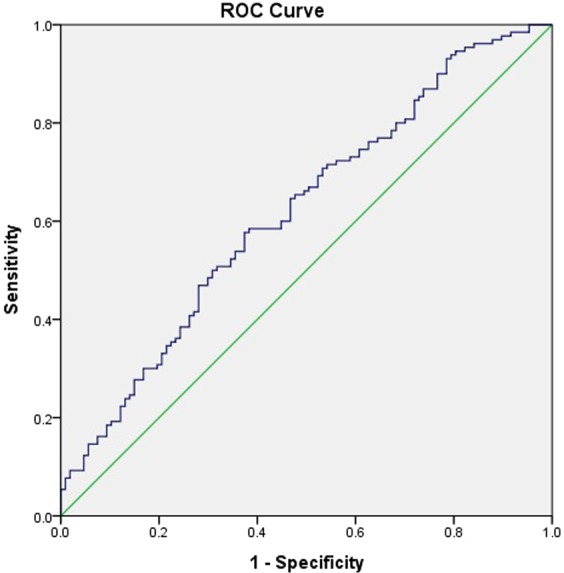
Receiving operator characteristic curve based on the sensitivity and specificity of the platelet-to-lymphocyte ratio (PLR).

The optimal cutoff value for the PLR, calculated by ROC analysis, was 181.24, with an area under the curve of 0.619 (*p* = 0.002) (Fig. 1). Clinical parameters were statistically compared between the high and low PLR groups, defined according to this cutoff value. The median age was 70 (range, 32–90) years in the high PLR group and 67 (range, 38–92) years in the low PLR group. The median OS was significantly different between the two groups (*p* < 0.001): 10.7 (range, 7.0–14.4) months versus 28.3 (range, 19.7–36.9) months in the high PLR versus low PLR group. There was no significant difference in PFS between the two groups. The proportion of males, ECOG PS, histologic features, proportion of ever smokers, tumor–node–metastasis stage, and first-line treatment modalities were not significantly different between the two groups, nor was the proportion of patients who underwent pleurodesis before or during anticancer treatment.

There were no significant differences in the mean white blood cell count, carcinoembryonic antigen, lactate dehydrogenase, or protein levels between the low PLR group and the high PLR group (8807.9 vs 9135; 114.2 vs 511.2; 496.4 vs 512.6; and 6.7 vs 6.6, respectively). The high PLR group showed significantly higher platelet level, PLR, lower albumin and hemoglobin levels than the low PLR group (329,630 vs 269,040, p < 0,001; 284.3 vs 135.0, p < 0.001; 3.6 vs 3.7, p = 0.005; and 12.7 vs 13.7, p < 0.001, respectively). The mean CRP level was higher in the high PLR group, although the difference did not reach statistical significance (p = 0.076).

### Overall survival

**Figure 2:**
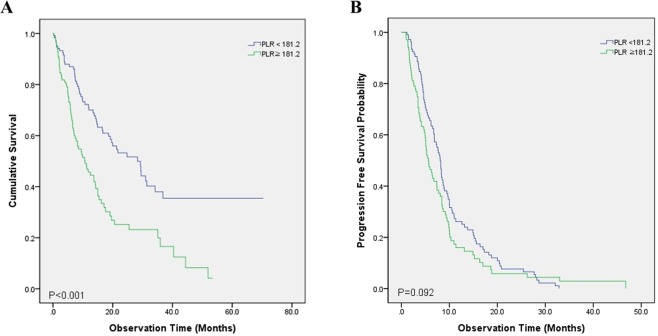
Kaplan–Meier survival curves after stratification by the PLR prior to treatment of non-small cell lung cancer (NSCLC), showing differences in (**A**) overall survival and (**B**) progression-free survival.

Univariate and multivariate analyses of survival are shown in Table 2. The following factors showed a significant association with shorter OS in the univariate analyses: male sex (*p* = 0.001), squamous cell histology (*p* = 0.019), not receiving active anticancer treatment (*p* < 0.001), lower hemoglobin level (*p* < 0.001), higher platelet count (*p* = 0.017), lower albumin level (*p* < 0.001), higher CRP level (*p* < 0.001), and higher PLR (*p* < 0.001) (Fig. 2A). These significant variables from the univariate analyses were entered into a multivariate analysis, which revealed the following as independent predictors of shorter OS: male sex (*p* = 0.010), not receiving active anticancer treatment (*p* < 0.001), lower hemoglobin level (*p* = 0.011), lower albumin level (*p* = 0.004), higher CRP level (*p* = 0.003), and high PLR (*p* < 0.001).

**Table 2:** Univariate analysis and multivariate analysis on OS and PFS in stage IV NSCLC with MPE.

Characteristics	OS				PFS			
	Univariate	Multivariate			Univariate	Multivariate		
	P	HR	95% CI	P	P-value	HR	95% CI	P
Age (≤70/>70)	0.069	1.077	0.725–1.599	0.714	0.915	1.017	0.723–1.431	0.924
Male	0.001	1.634	1.122–2.380	0.010	0.001	1.420	0.938–2.149	0.098
Smoking status (ever/never)	0.118				0.007	1.105	0.744–1.639	0.621
Histology (Adenocarcinoma/Squamous cell)	0.019	1.026	0.592–1.780	0.927	0.023	1.109	0.671–1.833	0.687
Driver mutation (Positive/Negative)	0.064				0.048	1.187	0.738–1.909	0.479
ECOG (0–1/≥2)	0.065				0.051			
T stage (1–2/3–4)	0.401				0.290			
N stage (0–2/3)	0.570				0.929			
M stage (M1a/M1b)	0.309				0.584			
Treatment (1^st^ line)								
Conventional chemotherapy	<0.001	—	—	<0.001		—	—	
Targeted therapy	0.159	0.770	0.497–1.191	0.240	0.023	0.739	0.447–1.220	0.237
Supportive care only	<0.001	12.80	5.44–30.13	<0.001	none			
Hemoglobin, g/dL (<12/≥12)	<0.001	1.905	1.157–3.134	0.011	0.001	2.169	1.363–3.452	0.001
Platelet, per uL (<300,000/≥300,000)	0.017	1.221	0.824–1.809	0.320	0.015	1.334	0.966–1.841	0.080
Albumin, g/dL (<3.1/≥3.1)	<0.001	1.873	1.227–2.860	0.004	0.043	1.050	0.681–1.617	0.826
CRP, mg/dL (<2.68/≥2.68)	<0.001	1.816	1.223–2.697	0.003	0.039	1.279	0.918–1.782	0.147
Platelet/ Lymphocyte (<181.2/≥181.2)	<0.001	2.161	1.457–3.204	<0.001	0.094			

BMI: body mass index; CI: confidence interval; CRP: c-reactive protein; ECOG: Eastern Cooperative Oncology Group; HR: hazard ratio; LDH: lactate dehydrogenase; OS: overall survival; PFS: progression free survival.

### Progression-free survival

In univariate analyses, the following variables showed significant associations with PFS: male sex (*p* = 0.001), history of smoking (*p* = 0.007), squamous histology (*p* = 0.023), poor ECOG PS (*p* = 0.048), not receiving active anticancer treatment (*p* < 0.001), lower hemoglobin level (*p* = 0.001), higher platelet count (*p* = 0.015), lower albumin level (*p* = 0.043), and higher CRP level (*p* = 0.039). However, a high PLR did not show a statistically significant association (*p* = 0.094) (Fig. 2B). All significant variables from the univariate analyses were entered into a multivariate analysis, which revealed that only a lower hemoglobin level was a significant risk factor for shorter PFS (*p* = 0.010) (Table 2).

### Wild-type EGFR and ALK NSCLC

**Figure 3:**
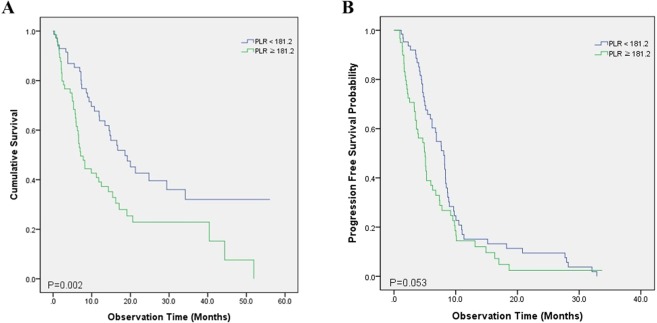
Kaplan–Meier survival curves after stratification by the PLR prior to treatment of NSCLC without driver mutations, evaluating differences in (**A**) overall survival and (**B**) progression-free survival.

Among the 142 patients with wild-type EGFR and ALK NSCLC, Kaplan–Meier analysis showed a significant difference in OS between the PLR groups (*p* = 0.002) (Fig. 3A). However, no significant difference in PFS was observed (*p* = 0.053) (Fig. 3B). The median OS was 27.1 months for the low PLR group and 16.2 months for the high PLR group. The median PFS was 8.0 months for the low PLR group and 5.1 months for the high PLR group.

Associations between patient factors and OS were evaluated. The following factors showed a significant association with shorter OS in univariate analysis: male sex (*p* = 0.005), squamous cell histology (*p* = 0.032), poor ECOG PS (*p* = 0.007), not receiving active anticancer treatment (*p* < 0.001), lower hemoglobin level (*p* < 0.001), lower albumin level (*p* < 0.001), higher CRP level (*p* < 0.001), and higher PLR (*p* = 0.003). These significant variables from the univariate analyses were entered into a multivariate analysis, which revealed that the following variables were independent predictors of shorter OS: poor ECOG PS (*p* = 0.047), not receiving active anticancer treatment (*p* < 0.001), lower hemoglobin level (*p* = 0.036), higher CRP level (*p* = 0.003), and higher PLR (*p* < 0.001) (Table 3).

**Table 3:** Univariate analysis and multivariate analysis on OS and PFS in 142 EGFR and ALK wild type stage IV NSCLC with MPE.

Characteristics	OS				PFS			
	Univariate	Multivariate			Univariate	Multivariate		
	P	HR	95% CI	P	P-value	HR	95% CI	P
Age (≤70/>70)	0.267	0.915	0.559–1.496	0.722	0.209	1.257	0.819–1.927	0.295
Male	0.005	1.864	0.965–3.598	0.064	0.091	1.323	0.865–2.022	0.197
Smoking status (ever/never)	0.032	1.020	0.544–1.914	0.951	0.078			
Histology (Adenocarcinoma/Squamous cell)	0.068				0.201			
ECOG (0–1/≥2)	0.007	2.551	1.013–6.422	0.047	0.021	1.805	0.782–4.167	0.166
T stage (1–2/3–4)	0.436				0.832			
N stage (0–2/3)	0.719				0.368			
M stage (M1a/M1b)	0.126				0.462			
Treatment (1^st^ line) (Conventional chemotherapy/Supportive care only)	<0.001	12.52	4.56–34.40	<0.001	—			
Hemoglobin, g/dL (<12/≥12)	<0.001	2.035	1.046–3.959	0.036	0.002	1.668	0.906–3.071	0.096
Platelet, per uL (<300,000/≥300,000)	0.197				0.035	1.504	0.987–2.292	0.057
Albumin, g/dL (<3.1/≥3.1)	<0.001	1.389	0.744–2.591	0.302	0.159			
CRP, mg/dL (<2.68/≥2.68)	<0.001	2.319	1.329–4.047	0.003	0.049	1.523	1.005–2.310	0.048
Platelet/ Lymphocyte (<181.24/≥181.24)	0.003	2.595	1.607–4.192	<0.001	0.053			

BMI: body mass index; CI: confidence interval; CRP: c-reactive protein; ECOG: Eastern Cooperative Oncology Group; HR: hazard ratio; LDH: lactate dehydrogenase; OS: overall survival; PFS: progression free survival.

In univariate analyses of PFS, the following variables showed significant associations with PFS: poor ECOG PS (*p* = 0.021), lower hemoglobin level (*p* = 0.002), higher platelet count (*p* = 0.035), and higher CRP level (*p* = 0.049). However, a high PLR did not show a statistically significant association (*p* = 0.053). All significant variables from the univariate analyses were entered into a multivariate analysis, which revealed a higher CRP level as the only significant risk factor for shorter PFS (*p* = 0.048) (Table 3).

## Discussion

The present study showed that a higher PLR was an independent predictor of shorter survival of stage IV NSCLC patients with MPE. This finding is consistent with previous studies that showed that an elevated PLR was an independent predictor of shorter OS of patients with NSCLC^9,13^. Several studies have shown that the PLR has prognostic value in NSCLC; however, the present study evaluated a multicenter lung cancer patient cohort, and to our knowledge, no previous study has evaluated the prognostic value of the PLR in stage IV NSCLC with cytologically and pathologically proven MPE. Identifying an association between the PLR and prognosis specific to NSCLC with MPE can be meaningful because NSCLC patients with MPE have distinct clinical characteristics. In lung cancer, MPE is associated with a poor prognosis^28^, and lung cancer patients with MPE have a decreased quality of life due to symptoms of dyspnea^29^.

Another novel finding of the present study is that a high PLR was associated with shorter survival of patients with wild-type EGFR and ALK NSCLC. The PLR, an important inflammatory marker with predictive value in NSCLC^14^, can also be used as a predictor of advanced NSCLC without major driver mutations. Targeted therapies such as EGFR and ALK tyrosine kinase inhibitors are typically used as first-line treatments for advanced NSCLC harboring EGFR and/or ALK mutations. While NSCLC patients with EGFR and ALK mutations show relatively improved survival compared with those with wild-type EGFR and ALK^22,23^, determining the prognostic value of the PLR in wild-type EGFR and ALK lung cancer would be meaningful.

An elevated PLR can be interpreted as a simultaneous increase in the platelet count and decrease in the lymphocyte count. Platelets in the tumor microenvironment release growth factors including thrombospondin, VEGF, platelet factor 4, and platelet-derived growth factor^30^. Promoted by these secreted growth factors, platelets in the tumor microenvironment promote sustained proliferative signals, metastasis, and evasion of immune detection^30,31^. Furthermore, thrombocytosis in the tumor microenvironment is related to chemoresistance^31^. On the other hand, lymphocytes play major anticancer roles^32^, and lymphocytopenia is associated with poor survival in cancer^33^. We assume that thrombocytosis and lymphocytopenia, which occur simultaneously, contribute to a shorter overall survival.

The cutoff of PLR determined in our study was 181.24. A previous study that evaluated the association between chemotherapy response and PLR used a cutoff of 152.6^7^, and another study evaluating surgically treated NSCLC patients used a PLR cutoff of 162^34^. Our cutoff value is relatively high compared with those of previous studies; however, we believe this is due to the different methods of selecting study patients. Thrombocytosis in the tumor microenvironment is associated with increased cancer activity and metastasis^30^, and all of the study patients in our study had stage IV NSCLC. Thus, thrombocytosis may have contributed to the relatively higher cutoff value.

The present study has several limitations. First, the median survival time was 15.4 months, which was significantly longer than the reported survival time of patients with stage IV NSCLC^20^. We believe that relatively higher proportions of female sex, never smokers, and positive driver mutations among the patients contributed to longer survival duration. In addition, patients with hematologic disease or significant infections were excluded. Second, the number of patients with EGFR and ALK wild-type NSCLC was small. We believe that the association between PLR and PFS might have differed if more study patients were enrolled.

## Conclusions

PLR is an independent predictor of shorter survival of patients with stage IV NSCLC, including those with wild-type EGFR and ALK. More research is needed to show how the PLR is related to cancer progression or treatment resistance in NSCLC and determine its underlying pathophysiology.

### Research involving Human Participants and/or Animals

For this type of study formal consent is not required.

### Informed consent

The requirement for informed consent was waived due to the retrospective nature of the study.
